# Combined spatial and frequency encoding for electrotactile feedback of myoelectric signals

**DOI:** 10.1007/s00221-022-06409-4

**Published:** 2022-07-25

**Authors:** Sara Nataletti, Fabrizio Leo, Jakob Dideriksen, Luca Brayda, Strahinja Dosen

**Affiliations:** 1 Cognitive Architecture for Collaborative Technologies Unit, Istituto Italiano di Tecnologia (IIT), Genoa, Italy; 2 Department of Informatics, Bioengineering Robotics, and System Engineering, University of Genoa, Genoa, Italy; 3 Acoesis S.R.L., Genoa, Italy; 4 Robotics, Brain and Cognitive Science Unit, Istituto Italiano di Tecnologia (IIT), Genoa, Italy; 5 Department of Health Science and Technology, Aalborg University, Ålborg, Denmark

**Keywords:** Closed-loop control, Myoelectric control, EMG feedback, Sensory feedback, Electrotactile stimulation

## Abstract

Electrotactile stimulation has been commonly used in human–machine interfaces to provide feedback to the user, thereby closing the control loop and improving performance. The encoding approach, which defines the mapping of the feedback information into stimulation profiles, is a critical component of an electrotactile interface. Ideally, the encoding will provide a high-fidelity representation of the feedback variable while being easy to perceive and interpret by the subject. In the present study, we performed a closed-loop experiment wherein discrete and continuous coding schemes are combined to exploit the benefits of both techniques. Subjects performed a muscle activation-matching task relying solely on electrotactile feedback representing the generated myoelectric signal (EMG). In particular, we investigated the performance of two different coding schemes (spatial and spatial combined with frequency) at two feedback resolutions (low: 3 and high: 5 intervals). In both schemes, the stimulation electrodes were placed circumferentially around the upper arm. The magnitude of the normalized EMG was divided into intervals, and each electrode was associated with one interval. When the generated EMG entered one of the intervals, the associated electrode started stimulating. In the combined encoding, the additional frequency modulation of the active electrode also indicated the momentary magnitude of the signal within the interval. The results showed that combined coding decreased the undershooting rate, variability and absolute deviation when the resolution was low but not when the resolution was high, where it actually worsened the performance. This demonstrates that combined coding can improve the effectiveness of EMG feedback, but that this effect is limited by the intrinsic variability of myoelectric control. Our findings, therefore, provide important insights as well as elucidate limitations of the information encoding methods when using electrotactile stimulation to convey a feedback signal characterized by high variability (EMG biofeedback).

## Introduction

An efficient human–machine interface (HMI) should not only allow the subject to convey commands to an external system but also provide sensory feedback from the system to the user to close the control loop. Closing the loop in neurofeedback and brain–computer interfaces, teleoperation, assistive robotics and virtual/augmented reality systems improves performance and facilitates the feeling of immersion and embodiment. Traditionally, many of these technologies have employed visual feedback because of its intrinsic resolution and high-information bandwidth (e.g., Hinterberger et al. 2004). However, visual feedback may not be adequate when the visual channel is already highly loaded by a complex task (Cincotti et al. 2007), in the case of visually impaired users (Loomis et al. 2012; Stronks et al. 2016; Leo et al. 2019), or whenever there is a need to restore missing somatosensory information, e.g., following traumatic injuries or neurological diseases, such as amputation or stroke (Antfolk et al. 2013b; Kita et al. 2013; Tzorakoleftherakis et al. 2015; Stephens-Fripp et al. 2018; Sensinger and Dosen 2020). In these cases, one can exploit the spatial extent of the skin to close the loop using touch. A particularly illustrative example comes from the prosthetics research, in which the data acquired from prosthesis sensors (e.g., grasping force) are transmitted to the participant through tactile stimulation of the forearm (D’Alonzo et al. 2015; Clemente et al. 2016; Markovic et al. 2018; Schofield et al. 2020).

One approach to activate the tactile sense externally is to use electrotactile stimulation, which involves the application of electrical current over the skin surface (Szeto and Saunders 1982; Szeto and Riso 1990; Clemente et al. 2016; Štrbac et al. 2016). This method has been tested in a wide range of practical applications, from telemanipulation and prosthetics to virtual reality (Kourtesis et al. 2022), and has been translated into commercial systems (BrainPort 2022; MyLeg 2022; Teslasuit 2022) and shown to integrate naturally into the motor control loop (Lewis et al. 2012; Akhtar et al. 2018; Gholinezhad et al. 2021). Importantly, designing an effective feedback interface requires defining an appropriate encoding scheme to map a feedback variable into a stimulation profile delivered to the skin. Ideally, the encoding should allow conveying the feedback information with high fidelity while enabling the stimulation to be clearly perceived and easily interpreted by the subject. The most straightforward approach employs a one-to-one mapping from the sensor domain to the stimulator. For example, feedback information can be transmitted to the user using a single stimulation channel and modulating one stimulation parameter (e.g., pulse width, amplitude, and frequency) proportionally to the measured variable (e.g., grasping force) (Chatterjee et al. 2008; Cipriani et al. 2008; Kita et al. 2013; Jorgovanovic et al. 2014). If a multichannel interface is available and the number of stimulator elements does not correspond to the number of input signals, then resampling methods may be used. For instance, the amplitude of the sensed signal can be conveyed by sequentially activating a single stimulator element within a linear array so that each location corresponds to a specific input value (i.e., so-called spatial modulation/encoding Saunders and Vijayakumar 2011; Witteveen et al. 2015). In general, spatial encoding is known to be easy to interpret, as the information is transmitted by activating spatially separate electrodes while the other parameters (intensity and frequency) remain constant (Szeto and Lyman 1977; Dosen et al. 2017; Nataletti et al. 2020). However, this scheme is also limited in resolution to a fixed number of information levels (each electrode codes one discrete level). On the other hand, continuous modulation in amplitude or frequency of a single channel has a much higher resolution, but the drawback is the limited ability of the subject to perceive and interpret the changes in the intensity/frequency (Szeto and Lyman 1977; Wilke et al. 2019b). Therefore, exploring the factors that can successfully improve the discrimination and interpretation of multiple concurrent tactile stimuli delivered over the body surface is of paramount importance.

The aim of the present research was to investigate novel sensory stimulation encoding schemes to improve the control of muscular activity in closed-loop control tasks. Specifically, able-bodied subjects performed a muscle activation-matching task with electrotactile stimulation representing the generated myoelectric signal. Using electromyographic (EMG) activity as biofeedback to promote upper limb rehabilitation has been explored in the past. For instance, the EMG biofeedback can indeed improve volitional activation of the hand muscles, and restore the hand function in persons with severe hand impairment due to chronic stroke (Wolf 1983; Moreland et al. 1994; Huang et al. 2006; Cordo et al. 2013). Similarly, experimental studies showed that this kind of biofeedback helps the modulation of myoelectric signals and thereby promotes the generation of more consistent commands for myoelectric prostheses, improving the quality of force control both in routine grasping and force steering tasks (Dosen et al. 2015b; Shehata et al. 2018b, a). Importantly, closed-loop control with EMG feedback is particularly suitable to investigate encoding methods because the feedback signal (EMG) is characterized by substantial variability, which makes translation into clear and intuitive stimulation profiles especially challenging. The EMG feedback has been typically delivered using discrete spatial coding (Dosen et al. 2015b; Schweisfurth et al. 2016; Tchimino et al. 2021). In this approach, the current level of muscle contraction (the “active” interval) was transmitted by activating a specific electrode or vibration motor within the array of stimulators circumferentially placed around the upper extremities.

The main novelty of our study, compared to previous works using only discrete coding (e.g., Schweisfurth et al. 2016; Tchimino et al. 2021), is the combination of discrete and continuous coding schemes to exploit the benefits of both techniques. We indeed hypothesized that this combination could potentially create an optimal EMG feedback interface that exploits the intuitiveness of the spatial technique and the higher resolution resulting from the continuous modulation of the stimulation parameters. In such combined encoding, each electrode still indicated the “active” EMG interval, but then, additionally, the stimulation frequency was also modulated to convey the momentary magnitude of the EMG within that interval, thereby providing more precise information about the muscle activation to the subject. We therefore evaluated whether a combined encoding strategy would improve the subject’s ability to generate and maintain the EMG within the desired interval compared to a simpler spatial coding. This hypothesis is in line with previous findings showing that using as many dimensions of a stimulus as possible to encode information effectively increases information transfer (Tan 1996; Novich and Eagleman 2015). There is indeed evidence that spatial (e.g., location) and temporal (e.g., frequency) features of tactile stimuli are processed by different receptors and afferents in the peripheral somatosensory system (e.g., Johnson 2001) as well as in partially different brain areas (e.g., Hegner et al. 2010; Yau et al. 2014), suggesting that these different features may be exploited in tactile feedback interfaces without confusing the subjects. We tested the subjects’ intrinsic capability to control their EMG using electrotactile feedback to directly compare the two encoding strategies. Note that such “virtual” online control without an actual physical system is a commonly used paradigm when the focus of the assessment is on the feedback and associated encoding schemes (Szeto and Lyman 1977; Erwin and Sup 2015).

Finally, the two encodings were also tested at two different feedback resolutions (i.e., low: 3 intervals and high: 5 intervals) using a different number of electrodes (i.e., 2 for the low resolution and 4 for the high resolution). This test was conducted because the resolution determines the number and width of the EMG intervals as well as the complexity of the feedback, both of which can critically affect the performance.

## Materials and methods

### Subjects

Eleven healthy subjects (29 ± 2 years, 10 males, 11 right-handed) with no known cognitive or tactile deficits took part in the experiment. The subjects signed an informed consent form before starting the experiment. The experimental protocol was approved by the ethical committee of Region Nordjylland, Denmark (approval number N-20 190 036).

### Experimental setup

**Fig. 1:**
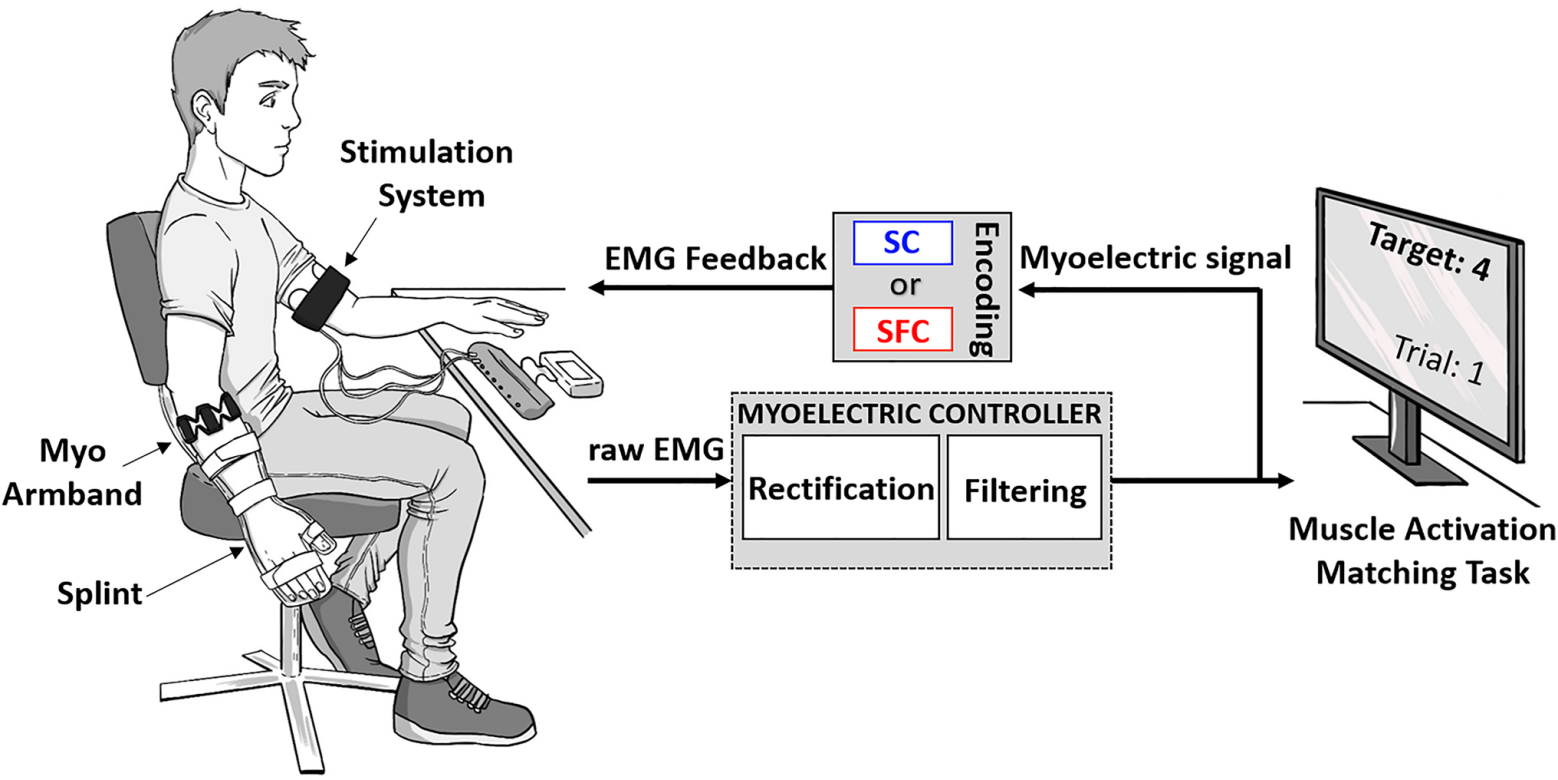
Block diagram of the online control using EMG feedback with two encoding schemes, spatial (SC) and combined spatio-frequency (SFC) modulation. The left side of the figure shows the subject, wearing an ic splint and the Myo Armband on the dominant forearm and the stimulation system with the electrode array wrapped circumferentially around the dominant upper arm. The stimulator was placed on a table connected to the computer via Bluetooth. The EMG recorded by the Myo Armband was rectified, low-pass filtered (0.5 Hz) and normalized to a percentage of MVC (50%). The myoelectric signal was sent to the computer, as well as back to the user through electrotactile feedback. The right side of the figure depicts the subject’s view during the experimental session, where a computer monitor displayed the tasks to be performed in the experiment (e.g., the target muscle activation interval and the trial number)

The experimental setup included the following components: (1) multichannel stimulator to deliver electrotactile feedback, (2) Myo Armband from Thalmic Labs for recording EMG signals, (3) a stiff orthopedic splint to secure the wrist and hand, thereby ensuring isometric muscle activation, (4) a 22″ computer monitor to provide task information to the subject, and (5) a laptop implementing a closed-loop control system (see Fig. 1).

The Myo Armband is a wireless wearable device that integrates eight dry, stainless-steel electrode channels that are equidistantly and circularly arranged around the circumference of the forearm. The armband was placed on the dominant forearm 2 cm distal to the elbow with the main module (marked by the logo) positioned on the dorsal side along the midline of the forearm. Only one channel was considered in the present study, namely, the one most active during isometric wrist flexion. The acquired EMG signals were sent via Bluetooth 4.0 to the host computer. The Myo armband recorded the EMG signals with a sampling rate of 200 Hz and was used previously for myoelectric control (Mendez et al. 2017; Visconti et al. 2018).

The electrotactile feedback was provided using a current-controlled multichannel stimulator prototype developed by Tecnalia Research and Innovation (Štrbac et al. 2016). The stimulation system generated current-controlled biphasic pulses with pulse amplitudes in the range of 0–100 mA (0.1 mA increments), pulse widths from 50 to 500 μs (10 μs increments) and pulse rates between 1 and 400 Hz (1 Hz increments). The unit integrated 12 stimulation channels with individually adjustable pulse width and amplitude, whereas the pulse rate was a global parameter common to all the channels. The delay between a positive and negative phase of the biphasic pulse was fixed by the construction of the stimulator to 1 ms. The stimulation parameters were set online from the laptop computer by sending simple text commands via Bluetooth.

Depending on the feedback resolution, we used two or four self-adhesive concentric electrodes (CoDe 501500, Spes Medica, Italy). The electrodes were equidistantly positioned around the nondominant upper arm of the subject, taking care to ensure that they were not located directly above the biceps since the stimulation sometimes induced involuntary contraction of this muscle. It remained impossible in 2 subjects to elicit a clear tactile sensation without slight muscle contraction, despite prior electrotactile calibration. These contractions were identified visually or through palpation and directly reported by the participant. Similarly, if the stimulation elicited an unpleasant or poorly localized (diffused or referred) sensation, the electrode position was slightly readjusted. A recent study by Guemann et al. (2019) demonstrated that the circular arrangement (transverse) elicited better spatial discrimination of stimuli than the linear arrangement (longitudinal to the upper arm axis). This may be due to the density and longitudinal orientation of the nerve fibers in the upper arm, which generates better transverse acuity because the stimulated area covers more separate fibers (Ross 1999). Electrotactile feedback was delivered to the contralateral arm to avoid interference with the EMG recordings. Each electrode consisted of an inner circle and an outer ring arranged in a concentric configuration. The diameter of the inner circle was 16 mm, while the outer diameter of the external ring was 42 mm with 5 mm of separation between the two; the thickness was approx. 1.5 mm (conductive pad: 1 mm, adhesive material: 0.5 mm). The concentric electrode is commonly used to provide electrotactile feedback since it ensures a localized and superficial current flow that elicits a focused sensation (Szeto and Saunders 1982; Szeto and Riso 1990; Štrbac et al. 2016).

During the experiment, the subject was seated in a comfortable chair, with the nondominant hand placed relaxed on a table and the dominant hand held vertically by the side of the body. The dominant forearm was placed into a stiff orthopedic splint to ensure that the subjects performed almost isometric muscle contraction. A monitor was positioned on the table approximately 50 cm from the subject and used to provide visual feedback when required (see “Experimental procedure”). The PC received recorded EMG signals and controlled the stimulation parameters. The online control loop was programmed in Simulink Desktop Real Time 2020a (MathWorks, USA) using a flexible test bench for the assessment of closed-loop control (Dosen et al. 2015a).

### Myoelectric control

The acquired EMG signals were full-wave rectified and smoothed using a second-order low-pass Butterworth filter with a cutoff frequency of 0.5 Hz to produce a smooth control input (myoelectric signal). A recent study demonstrated that the low cutoff frequency allows the subjects to exploit the feedback more effectively and thereby improve the online control of the EMG signal, increasing the performance of EMG biofeedback in routine grasping (Tchimino et al. 2021). Nevertheless, this could be a limitation in the activities that require particularly fast reaction (e.g., catching an object).

Before starting the online myocontrol task, myoelectric control was calibrated for each subject individually. The baseline EMG and the maximal voluntary contraction (MVC) were measured and used to adjust gains and dead zones. To obtain the baseline, subjects were asked to keep the dominant arm completely relaxed for 10 s. After that, they performed a 5-s long maximum wrist flexion, and the maximum value of the generated myoelectric signal was recorded. This was repeated three times, and the averaged value was adopted as the MVC. The myoelectric signal was then normalized so that the EMG range between the baseline and 50% MVC was linearly mapped between 0 and 100%, also following the recommendations in (Tchimino et al. 2021). When the subjects relaxed their muscles, the small muscle activations still present were filtered out by defining a dead zone (< 5% of the normalized EMG). The subthreshold signals resulted in zero control input and no stimulation.

### Electrotactile EMG feedback

During the online myocontrol task, the myoelectric signal was transmitted to the subject as feedback information using electrotactile stimulation. To establish the stimulation amplitude, *detection thresholds* (DT) were identified for each electrode using the method of limits (Kingdom and Prins 2009). The stimulus intensity was increased in steps to find the amplitude at which the subject first reported that they felt the stimulation. The pulse width and frequency were fixed at 250 μs and 4 Hz (the lowest value), respectively, while the amplitude was automatically increased every 1.5 s, with a step size of 0.1 mA, starting from 0.5 mA (subthreshold amplitude). The pulse amplitude was set to 2 × DT and kept constant during the experiment. Before commencing with the experiment, the subjects received a burst of stimulation at this amplitude and the highest frequency (60 Hz) to ensure that the stimulation was comfortable in all cases.

**Fig. 2:**
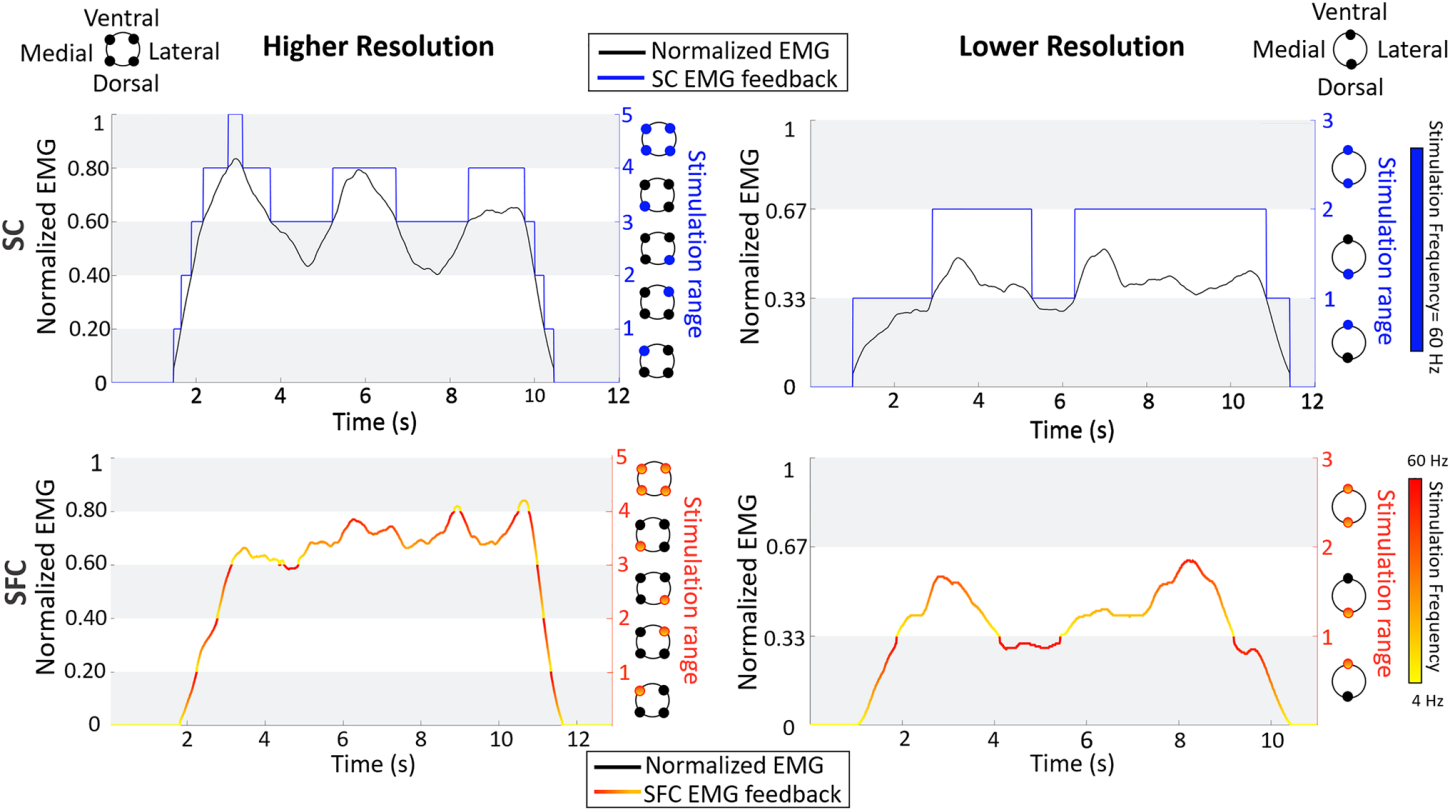
The electrotactile EMG feedback delivered to the subject for the two feedback resolutions (higher and lower) and codes (SC and SFC). The normalized myoelectric signal was divided into 5 and 3 intervals (white and grey stripes) in the higher (left panels) and lower (right panels) resolution, respectively. In SC (upper panels, in blue), the momentary interval in which the generated myoelectric signal (black line) resided was indicated by activating different electrodes with a fixed frequency of 60 Hz. In SFC (lower panels, shades of red), the spatial activation was supplemented with frequency modulation (4–60 Hz) conveying the magnitude of the myoelectric signal within the “active” interval. Note that in the SFC panels, the line still indicates the myoelectric signal, but the color represents the frequency of stimulation associated with the signal value (within the interval), as specified by the color map

The electrotactile EMG feedback conveyed to the subject the momentary level of the generated myoelectric signal, thereby augmenting the information that the subjects received through natural muscle proprioception (sense of contraction strength). As explained in the experimental procedure section, the subjects were instructed to employ online EMG feedback to adjust their muscle activation within the target interval shown on the computer screen. Practically, the subjects increased their contraction strength until they felt that the correct electrotactile code (specific electrode and frequency) was activated. Two coding schemes depicted in Fig. 2 were designed to transmit the EMG information, one using spatial coding (SC) and the other using combined spatial and frequency coding (SFC). The normalized myoelectric signal was divided into equal intervals, which were then mapped to feedback codes. In SC, each interval was associated with a single electrode. The absence of stimulation indicated the dead zone interval. When the magnitude of the myoelectric signal generated by the subject entered a specific interval, the respective electrode started stimulating. Therefore, the currently active electrode indicated the muscle activation level (“active” interval) to the subject. For instance, when it was within the first interval, only the first electrode was activated; when it was within the second interval, only the second electrode was active, and so on. Finally, to indicate that the maximal interval was reached, all electrodes were simultaneously activated. The stimulation frequency and pulse width were fixed at 60 Hz and 250 μs, respectively.

In SFC, the spatial code used in the SC was enriched with frequency modulation. Therefore, the SFC is a combined coding where the information on the generated myoelectric signal is conveyed by changing both the active electrode (location) and frequency. Specifically, the current interval of the generated myoelectric signal was indicated by the activated electrode (as in the SC), while the level of the signal within that interval was determined by modulating the stimulation frequency. The higher frequency corresponded to the higher EMG signal within the respective interval. Therefore, the lower (higher) frequencies indicated that the subject was near the lower (upper) limit of the particular interval. The pulse width was fixed at 250 μs, while the stimulation frequency was modulated between 4 and 60 Hz. We used this range of frequencies since two further studies on electrotactile feedback used encoding frequencies < 80 Hz (Anani and Körner 1979; Dideriksen et al. 2020). Frequencies above 100 Hz are more difficult to differentiate since they produce fused and similar sensations and are more prone to adaptation (Szeto and Lyman 1977; Szeto and Saunders 1982; Valle et al. 2018). Therefore, compared to SC, in SFC, the subjects received more precise information on the magnitude of the myoelectric signal (SFC: interval + level within the interval, SC: interval only). The hypothesis was that this additional information would allow the subject to decrease the variability of the myoelectric signal and better maintain the signal within the target interval.

Each coding scheme was tested using two different feedback resolutions, i.e., the number of equispaced intervals into which the normalized myoelectric signal was divided. Specifically, we used 5 intervals (I*n*) for higher resolution (I1: 1–20%, I2: 20–40%, I3: 40–60%, I4: 60–80%, and I5: 80–100%) and 3 intervals for lower resolution (I1: 1–33%, I2: 33–66%, and I3: 66–100%). A similar number of intervals was used previously in the literature when transmitting force and EMG feedback (Antfolk et al. 2013a; Kita et al. 2013; Jorgovanovic et al. 2014; Raspopovic et al. 2014; De Nunzio et al. 2017; Markovic et al. 2017; Wilke et al. 2019a).

The five (higher resolution) and three (lower resolution) intervals were mapped using *N* = 4 and *N* = 2 electrodes, respectively. Feedback mapping is presented in Fig. 2; the interval I*n* was encoded by the activation of the *n*th electrode (where *n* = 1,..., and *N* is the electrode number), while the last interval I*n* (where *n* = *N* + 1) was indicated by the simultaneous activation of all the electrodes. This mapping was the same in SC and SFC, but in SFC, the frequency of the active electrodes was additionally modulated to convey the magnitude of the myoelectric signal within the respective interval. In both resolutions, the electrodes were equidistantly placed around the circumference of the upper arm, starting by positioning the first electrode on the dorsal side and moving counterclockwise to place the remaining electrodes (see the sketches in Fig. 2).

### Experimental procedure

Each subject took part in two experimental sessions, with 10 days between them. Higher resolution feedback was evaluated in the first session and lower resolution in the second. The sessions were not randomized because the aim was to compare the two coding schemes (and not the resolutions). Nevertheless, we ensured that the two sessions were separated long enough to minimize the training effect. In each session, the two different electrotactile encodings (SC and SFC) were tested, and each test lasted approximately 2 h. The order of the two encodings was counterbalanced across subjects. The session comprised two phases common to both encoding methods (feedback interface training and myocontrol training) and two additional phases specific to each coding scheme (closed-loop training and muscle activation-matching task).

First, the subjects were familiarized with the stimulation sensations for a few minutes. All electrotactile stimuli (5 or 3) coding the intervals of the feedback variable were delivered to the subject, from the lowest to the highest, so that the subjects experienced the full set of possible spatial electrotactile patterns. To train the ability to discriminate the patterns, *reinforced learning* was employed. During this phase, the patterns were delivered randomly for 1 s, after which the subject was asked to report the interval (from 1 to 5 or 1 to 3) conveyed by the electrotactile feedback. If the subject answered correctly, the experimenter moved to the next pattern. If the subject answered incorrectly, the experimenter indicated the correct interval verbally. Each interval (pattern) was presented at least three times, and this phase was repeated until the recognition rate was 100%. This phase lasted not more than ten minutes.

Next, to train the subject in controlling the myoelectric signal, visual feedback about the generated EMG was provided. The visual feedback was displayed as a bar on the computer screen, where the height of the bar was proportional to the normalized myoelectric signal. The area of the bar was divided into the same intervals that were conveyed using electrotactile EMG feedback. Therefore, the subject could observe how their muscle contraction modulated the generated signal, and they were asked to reach and maintain the signal within the indicated intervals on the bar.

After having trained the myoelectric control and electrotactile stimulation separately, the EMG control and electrotactile feedback were then combined. Here, visual feedback (bar plot) was also shown while the subject received the stimulation. The subjects, therefore, learned the task they should perform as well as the mapping between the visual EMG feedback and the corresponding electrotactile feedback. The subject was given a few minutes of training to be acquainted with the myoelectric control and to train the feedback scheme interpretation.

After a short break, the subject performed the main experiment comprising a sequence of *muscle activation-matching tasks*. In each trial, a random target interval (1–4 for higher resolution and 1–2 for lower resolution) was presented. Subjects were instructed to increase the myoelectric signal as fast as possible to reach the indicated target interval and then to try to maintain the signal within that interval for 7 s. Once the subject reached the target interval for the first time within a trial, a timer started, and after 7 s, a message “trial completed, now relaxing!” appeared on the screen to indicate the end of the trial, and the subjects relaxed their muscles. After a break of 3 s, the next trial started. The trial number and target EMG intervals were shown on the computer screen. Each target interval was presented for 20 trials, leading to a total of 80 trials for higher resolution and 40 trials for lower resolution. These trials were split into two blocks with a 5-min break in between. Importantly, during this phase, the subjects received only tactile feedback about the myoelectric signal (the visual bar was removed from the screen). We excluded the highest interval (fifth or third depending on whether the higher or lower resolution was used) since it was not informative with respect to the subject’s ability to control the myoelectric signal. We observed in a pilot study that reaching and staying within the highest interval was trivial, as it was sufficient to perform the strongest contraction and saturate the myoelectric signal to the maximum value.

### Data analysis

Five outcome measures were examined to evaluate the performance in the muscle activation-matching task: the time to reach the target interval (rising time), the undershooting and overshooting rates, the absolute deviation, and the variability of the myoelectric signal.

The rising time was measured from the moment when the signal crossed the dead zone until the target interval was reached for the first time (i.e., crossing the lower threshold). This measure was used to examine how fast the subject could reach the target interval. The over/undershooting rate was used to assess the stability of control and was defined as the percentage of time the signal was over/under the desired interval after reaching that interval for the first time. The absolute deviation was calculated as the absolute value of the average difference between the myoelectric signal and the center of the target interval in each trial. The variability of the myoelectric signal was defined as the standard deviation of the signal. The variability evaluated the *precision* of control, i.e., how consistent the subjects were in maintaining a specific signal level, regardless of how close they were to the center of the target interval, while the absolute deviation characterized the *accuracy* of control. Ideally, the EMG feedback would assist the subject in reaching the desired level quickly (small rising time) and then maintaining the myoelectric signal within the interval (small over(under)shooting rate). Ideally, the subjects would keep the signal close to the middle of the target interval while decreasing its variability, thereby providing a safety margin to prevent crossing the interval boundaries. Note that both variability and absolute deviation are dimensionless outcomes since they are the standard deviation and the absolute value of the average difference of a normalized variable, respectively. Both of these variables were measured in the phase where the subject had to maintain the contraction (7 s).

We used the Shapiro–Wilk test to assess the normality of the data distributions. Most distributions violated the assumption of normality. Hence, we used non-parametric tests, namely, Friedman tests, as an alternative to the repeated-measures ANOVA and, when needed, Wilcoxon signed-rank tests for post hoc pairwise comparison; all *p* values were corrected with false discovery rate correction using the Benjamini–Hochberg method (Benjamini and Hochberg 1995).

To test our first hypothesis, that the subject’s ability to stay inside the target interval was improved by adding frequency modulation, we applied Wilcoxon tests to all outcome measures for both resolutions with the feedback coding scheme as a within-subjects factor (interaction between feedback coding and resolution).

It is well known that EMG noise is dependent on signal size; hence, larger control signals (from stronger contractions) are more variable and difficult to control (Clancy et al. 2002). Therefore, to investigate how the target interval amplitude modulated the subjects’ performance with EMG feedback, we calculated the mean values of each outcome measure per interval for both resolutions regardless of the electrotactile code; after that, we applied Friedman tests to all outcome measures with the target interval as a within-subjects factor (interaction between target interval and resolution). Moreover, to evaluate the strength of the obtained results in terms of the magnitude of the difference in the mean scores of the groups, we estimated the effect size r for each Wilcoxon signed-rank test using the formula *r = *$\frac{z}{\sqrt{n}}$. For the interpretation of the effect sizes, we followed Cohen’s guidelines (Cohen 1988): small, medium, and large effects correspond to *r* > 0.1, *r* > 0.3, and *r* > 0.5, respectively.

Statistical analysis was conducted in Python (Python Software Foundation). The threshold for statistical significance was set to *p* < 0.05. The results in the following sections are reported quantitatively as median (M) and interquartile range (IQR), i.e., M{IQR}, where IQR = Q75–Q25, and Q75 and Q25 are the 75th and 25th percentiles of the data distribution, respectively.

## Results

**Fig. 3:**
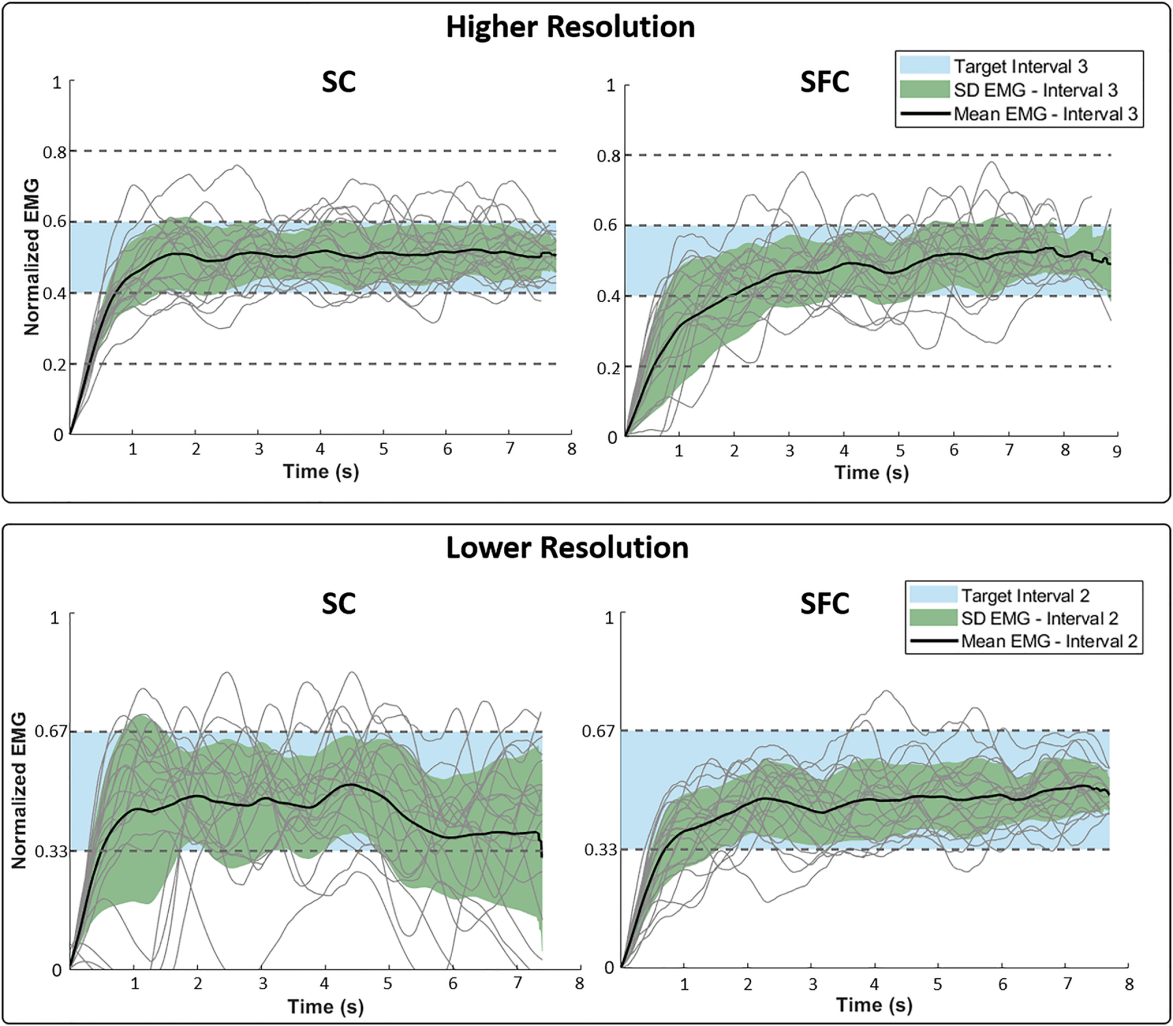
Representative myoelectric signals generated by one subject using higher resolution (upper panel) and lower resolution (lower panel) feedback and two coding schemes (SC—left plots and SFC—right plots). The task for the subject was to use the feedback to reach and maintain the indicated target interval (blue shading). The generated signals are shown using light lines (for a total of 20 trials), while the solid black lines indicate the mean values and the shaded green area is ± 1 standard deviation. The SFC coding seems to improve the performance (decreased variability and overshooting/undershooting) but only for the lower resolution feedback

Representative profiles of myoelectric signals generated by one subject are shown in Fig. 3. Across all coding schemes, the subject increased the EMG signal up to the desired interval (rising segment) and then maintained the contraction for 7 s (plateau) until the end of the trial. Overall, the myoelectric signals demonstrated that the subject successfully modulated the muscle contraction using online electrotactile feedback. Once the subject reached the target interval (indicated as a light blue patch), the generated EMG signals were usually within the target interval with occasional excursions to one interval above or below the target. Furthermore, the fact that this subject needed only a few seconds to reach the desired interval suggests that the feedback was intuitive. By comparing the performance across the coding schemes, it seems that SC and SFC enabled the subject to stay within the target interval equally well in the higher resolution case. In the lower resolution case, however, it seemed that the additional frequency modulation of the SFC scheme facilitated the subject’s performance. Indeed, the generated myoelectric signal was visibly less variable using SFC compared to SC. Furthermore, the numbers of overshooting and undershooting events were considerably diminished, while the time to reach the target interval seemed to be comparable.

**Fig. 4:**
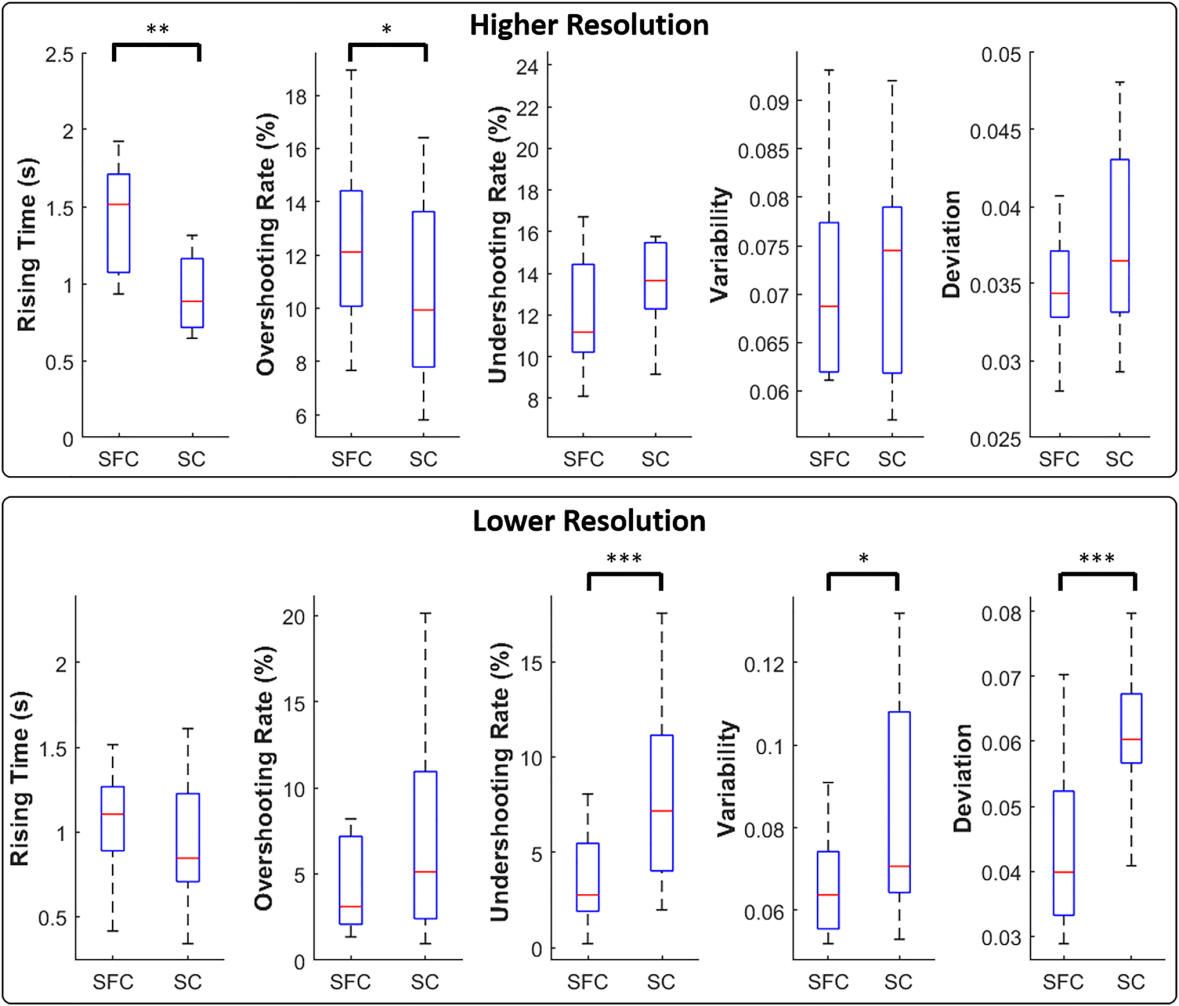
Summary performance across the two electrotactile codes (SFC and SC) for higher (upper panel) and lower resolution (lower panel). From left to the right, the boxplots show the medians and 25 and 75 percentiles of rising time, overshooting rate, undershooting rate, variability and absolute deviation for each electrotactile code. Asterisks indicate statistically significant differences. *p < 0.05; **p < 0.01; ***p < 0.001

The summary results (M{IQR}) of the outcome measures *across the two electrotactile codes* are shown in Fig. 4 (upper panels). As indicated by the representative data (Fig. 3), SFC had a positive effect on performance in the lower resolution case only. With the higher resolution, SFC was characterized by significantly higher rising time (1.5{0.6} s) than SC (0.88{0.4}) (*T* = 1.0, *p* < 0.01, *r* = 0.77), and the subjects spent more time above the upper limit of the target interval (12{4}%) compared to SC (10{5}%) (*T* = 7.0, *p* < 0.05, *r* = 0.66).

The performance *across target intervals* showed that the variability of the generated EMG signal increased steadily and significantly (Friedman test: *χ*^2^ = 29.7, *p* < 0.001) with the target EMG level (I1: 0.049{0.01} to I4: 0.093{0.03}). A significant increase was also observed for the absolute deviation (*χ*^2^ = 23.7, *p* < 0.001¸ I1: 0.054{0.02} to I4: 0.085{0.03}), rising time (*χ*^2^ = 11.6, *p* < 0.001, I2: 0.5{0.2}s to I4: 1.6{1}s), and undershooting rate (*χ*^2^ = 26.6, *p* < 0.001, I1: 3{4}% to I4: 25{10}%). The overshooting rate, however, was not significantly affected by the target interval.

Contrary to what we found for the higher resolution, the variability and the absolute deviation for the lower resolution feedback decreased significantly (variability: *T* = 51.0, *p* < 0.05 and *r* > 0.7; deviation *T* = 9.0, *p* < 0.001 and *r* > 0.8) with SFC (variability: 0.064{0.02}; absolute deviation: 0.040{0.02}) compared to SC (variability: 0.071{0.04}; absolute deviation 0.060{0.01}).

In addition, the subjects spent significantly more time (*T* = 14.0, *p* < 0.001, *r* = 0.84) undershooting the target level when using SC (7.1{7}%) compared to SFC (2.7{3}%).

The performance *across target* interval*s* exhibited the same trend as that obtained for the higher resolution feedback. Specifically, variability (*T* = 46, *p* < 0.001 and *r* = 0.7 interval 1: 0.059{0.03}, interval 2: 0.074{0.03}), absolute deviation (*T* = 44, *p* < 0.01 and *r* = 0.7; interval 1: 0.069{0.03}, interval 2: 0.087{0.02}) and undershooting rate (*T* = 26.6, *p* < 0.001 and *r* = 0.8; interval 1: 2.9{5}%, interval 2: 8.8{6}%) increased significantly from interval 1 to interval 2.

## Discussion

In this study, we compared the effectiveness of two encoding schemes (spatial or SC and combined or SFC) to deliver EMG feedback using electrotactile stimulation while subjects were asked to generate and maintain the myoelectric signal within an indicated target interval. In the case of spatial coding, the electrotactile feedback conveyed information only about the current discrete interval of the generated myoelectric signal, while in the combined coding approach, it also indicated the momentary magnitude of the signal within that interval using proportional frequency modulation. We hypothesized that the additional information provided by the combined approach would facilitate more accurate closed-loop control. However, this hypothesis was only partially confirmed, as the SFC feedback improved the performance only when the feedback resolution was low. At the higher resolution, the SFC was even detrimental to the performance, as discussed as follows.

For the higher resolution feedback (5 intervals), the two encoding strategies performed similarly with respect to undershooting rate, variability and absolute deviation, while the overshooting rate and the rising time were both lower in SC than in SFC. These results suggest that the extra information provided by the SFC was more a distraction than a useful addition to the feedback, as the subjects became slower without performance improvement. As there were more intervals in the higher resolution feedback, the same range of frequencies (4–60 Hz) was mapped to a smaller range of the normalized myoelectric signal (20% in higher versus 33% in lower resolution feedback). This made the task more difficult due to the intrinsic variability of the EMG, while the frequency modulation was more dynamic. It is therefore possible that the subjects had difficulties in perceiving and interpreting larger and faster changes in frequency and/or they were overwhelmed by the intrinsic ‘noise’ of the EMG. In the latter case, even if the subjects were able to correctly interpret the frequency mapping, it might not have been possible for them to exploit this information to improve the myoelectric control.

When the feedback resolution was lower, however, the subjects were able to exploit the richer information delivered by the frequency modulation within the intervals, at the cost of more gross information (larger ranges) provided by the spatial disposition of the electrodes. SFC resulted in a lower undershooting rate, absolute deviation, and variability than SC. With lower resolution, the feedback intervals became wider, which allowed the subjects to effectively exploit the frequency modulation. They were more conscious about their position within the target interval and were thereby able to maintain their myoelectric signal closer to the center of the interval. We speculate that during familiarization, they learned the frequency corresponding to the center of the interval: then, during testing, they attempted to achieve and maintain that frequency. Given the larger intervals, the frequency variation for equal EMG signal change was more gradual and noticeable than in the high-resolution feedback. It is possible, however, that appropriate training may also lead to better performance using SFC in the higher resolution case. Several studies have demonstrated that the ability to discriminate multiple spatial channels (Witteveen et al. 2012; Štrbac et al. 2016) and frequency levels (Anani et al. 1977) can substantially improve after training. The results of Štrbac et al. (2016) showed that an able-bodied subject could achieve high performance in recognizing multiple pads after prolonged training. Similar improvements in performance after long-term training might be expected for frequency discrimination, as shown by Riso et al. (1989) and Anani et al. (1977). Moreover, previous studies showed that specific training regimes using visual or tactile feedback of hand force or finger pressure improve force control and reduce its fluctuations in patients with upper extremity impairments (Quaney et al. 2010; Kita et al. 2013; Bouwsema et al. 2014).

Regardless of the feedback resolution and coding, the performance of myoelectric control decreased consistently with higher target intervals (increased variability, absolute deviation and undershooting rate). It is well established that EMG variability increases with contraction intensity (Harris and Wolpert, 1998; Parker et al. 2006; Campbell et al. 2020), which makes the task more difficult, and it seems that neither more informative coding nor lower feedback resolution was enough to prevent the consequent decrease in performance (variability at lower resolution: I1: 0.059 to I2: 0.074; higher resolution: I1: 0.049 to I4: 0.093). Interestingly, the overshooting rate remained almost unaffected across the intervals. This is probably because the subjects tended to remain nearer the lower limits of the EMG interval, where the signal modulation is by nature more controllable while limiting muscle fatigue. It is well recognized that effort represents one of the universal cost functions that govern the control of movements. In particular, humans tend to be accurate enough while minimizing effort and variability (Todorov and Jordan 2002; O’Sullivan et al. 2009; Haith et al. 2012).

Noninvasive feedback is most often nonsomatotopic and not matched in modality; hence, its interpretation requires, at least initially, some cognitive effort and training. Importantly, the feedback in the present study was implemented using the combined encoding approach, which has been shown to be easy to interpret (Dosen et al. 2017). However, in that study, the frequency modulation was also discrete, while the feedback conveyed a stable signal (prosthesis grasping force). In addition, the exact placement of the electrodes is not critical for the presented approach as long as the electrodes are separate enough to be easily discriminated. Nevertheless, it remains to be investigated how the use of such feedback in a more practical task (e.g., including a prosthesis in the loop), when the subjects need to divide their attention, would affect the feedback interpretation and cognitive effort as well as if the latter could be decreased with training. As shown in a recent study by Gholinezhad et al. (2021), humans can subconsciously process electrotactile stimulation. Not only did the presence of electrotactile stimulation not impair the perception of natural feedback, but it actually enhanced the task-relevant natural input and improved the overall state estimate. Nevertheless, a possible drawback of electrotactile stimulation is its tendency to cause adaptation, which is particularly pronounced when the stimulation is prolonged and constant (Szeto and Saunders 1982). However, the feedback delivered in the present study was dynamic, as the stimulation was delivered to different sites with continuously changing frequency. Dynamic stimulation is less prone to adaptation (Buma et al. 2007), and none of the subjects in the present experiment reported that they had difficulties perceiving the feedback.

In summary, this study compared the effectiveness of different noninvasive EMG feedback coding schemes for reducing the variability of the myoelectric signal and the deviation from a specific target interval in an abstract task. The results showed that with spatial encoding, the addition of frequency modulation implies a significant benefit in spatially encoded feedback with low spatial resolution. In contrast, with higher resolution, this solution brings no advantage and might even worsen the performance. These findings have important implications for the understanding and design of the optimal encoding system using electrotactile stimulation and reveal the limitations of different approaches to facilitate closed-loop “tactile” control with a highly variable feedback signal (EMG).

## Data Availability

The data that support the findings of this study are available from the corresponding author upon reasonable request.
